# Functional quality of optimized peach‐based beverage developed by application of ultrasonic processing

**DOI:** 10.1002/fsn3.1227

**Published:** 2019-10-14

**Authors:** Saira Sattar, Muhammad Imran, Zarina Mushtaq, Muhammad Haseeb Ahmad, Melvin Holmes, Joanne Maycock, Muhammad Imran Khan, Adeela Yasmin, Muhammad Kamran Khan, Niaz Muhammad

**Affiliations:** ^1^ Faculty of Life Sciences Institute of Home and Food Sciences Government College University Faisalabad Pakistan; ^2^ School of Food Science and Nutrition University of Leeds Leeds UK; ^3^ Department of Mathematics & Statistics Faculty of Sciences University of Agriculture Faisalabad Pakistan; ^4^ National Agriculture Education College Kabul Afghanistan

**Keywords:** antioxidant activity, bioactive compounds, organic acids, pasteurization, ultrasonic processing

## Abstract

The influence of thermal treatment (at 90°C for 10 min) and sonication (at 20 kHz and 130 W for 30, 60, and 90 min on room temperature) on the physicochemical properties, bioactive compounds, antioxidant activity, and organic acids of fresh formulated functional peach beverage was investigated. The results indicated that conventional pasteurization and sonication treatment did not show any significant changes in pH value and Brix amount of juice, and however, a rise in cloud value was observed under all processing conditions. The thermal treatment caused the decrement in total phenolic content (TPC), total flavonoid content (TFC), antioxidant activity (assessed by diphenyl dipicryl hydrazyl (DPPH), ferric ion reducing antioxidant power (FRAP) and 2,2'‐azino‐bis(3‐ethylbenzothiazoline‐6‐sulfonic acid) (ABTS)), and organic acids of juice, whereas sonication treatment for 90 min increased maximum the activity of bioactive compounds (TPC: 600.61 µg/100 ml; TFC: 177 µg CE/100 ml), antioxidants (DPPH: 51.87%; FRAP: 506.13 µmol Trolox/L; ABTS: 1,507.375 µmol Trolox/L), and organic acids (malic acid: 998; citric acid: 128; oxalic acid: 145; shikimic acid: 63 µg/100 ml) as compared to other treatment conditions and control. Multivariate data analysis was done by principal component analysis as it identifies patterns in data by comparing data sets which is further expressed based on their similarities and discriminations, respectively.

## 1 INTRODUCTION

Beverages with esteemed quality are in continuous demand by the consumers. Therefore, the use of innovative technologies in minimizing the losses of beverage quality during processing is in great consideration among the processors (Abdullah, 2012). Traditional processes depend on heat to eradicate or inactivate foodborne pathogens (such as microorganisms, viruses, and parasites) to ensure food is safe to eat. For several foods, heating is a good method to treat contamination (Chen, Yu, & Rupasinghe, 2013). The shelf‐life of beverages is traditionally achieved by using thermal processes. The effects of thermal processing regarding inactivation of microbes for food preservation is reported by many researchers, however, it may also cause the destruction of desirable nutrients and sensory properties of the food (Mosqueda‐Melgar, Raybaudi‐Massilia, & Martín‐Belloso, 2012).

Presently, ultrasound is among the emerging novel processing technologies that is vigorously used as a food processing method due to its nonthermal attribute which causes less modification in food products like tenderization of meat (Hu et al., 2018) as compared to thermal processing. Ultrasound, based on cavitation phenomenon, is defined as sound waves having frequency that exceeds the limit of the human ear (~20 kHz). High pressure causes the generation of cavitation bubbles which collapse violently in the succeeding compression cycle of propagated sonic wave (Valdramidis, Cullen, Tiwari, & O'Donnell, 2010). The effect of cavitation in beverage treatments relies on several factors including frequency and power of sonication waves, temperature, and characteristics of the treatment liquid (Belgheisi & EsmaeilZadeh Kenari, 2019). Studies from the past few years have confirmed that sonication is a moderate food preservation technique competent in inactivating deteriorative microbes and enzymes, and without any modification to physicochemical, nutritional, and sensorial properties providing safe and fresh‐like products (Petruzzi et al., 2017).

The presence of nutritional, antioxidant, and bioactive compounds in fruit juices proved them the best example of functional beverages (Ogundele, Awolu, Badejo, Nwachukwu, & Fagbemi, 2016). These compounds enhance consumer health and improve the taste and visual appearance to vegetables and fruits. It also minimizes cancer and cardiovascular risks by stabilizing the free radicals (carcinogenic) manufactured during metabolism process (Khouloud, Abedelmalek, Chtourou, & Souissi, 2018). The physiological and remedial benefits of peach juice make it a potential drink to act as a functional beverage in beverage industry. Peach juice is a major source of different phytochemicals, which are also responsible for the color of the juice (Rojas, Leite, Cristianini, Alvim, & Augusto, 2016). Other than bioactive and functional compounds, it contains carotenoids, thiol, and vitamins A, C, and E along with a bunch of organic acids (citric acid, malic acid, shikimic acid, and quinic acid) (Wolfe et al., 2008). Previously, the functional beverages were developed either by just mixing of different fruit juices or by treating the single composite juice with novel technologies. The current work emphasizes on the development of functional beverage superior than previous studies as it presents the mixture of different juices for the integrated effect among various bioactive compounds as well as treatment with novel technology of ultrasound to retain maximum bioactivity of these compounds. Therefore, the present study was carried out to find out best ultrasound treatment conditions on the quality parameters of peach functional beverage that is incorporated with plum juice and sugar solution.

## 2 METHODOLOGY

### 2.1 Collection of raw materials

The fruits (peach and plum) for the formulation of juices were collected from “Abaseen local shop” of Leeds, UK, which were then washed, disinfected, and air‐dried. The fruits were then deseeded, and pulping was done using household juicer (2,050 *g*, 4 min). The juices were than filtered and mixed in optimized proportion with sugar solution which was selected as a best sensory score in previous study, that is, 72% (Peach): 25% (Plum): 3% (Sugar solution) of our research group (Sattar et al., 2019). The samples were then kept into refrigerator until further analysis.

### 2.2 Conventional pasteurization

A 100 ml of fresh juice kept in falcon tubes was heated for 10 min at 90°C using a thermostatic water bath (Grant, model VFP), which was then instantly shifted to ice water so that juice can attain the temperature of 20°C, and the prepared juice samples were then stored in freezer at (−18°C) until further analysis (Tembo, Holmes, & Marshall, 2017a, 2017b) .

### 2.3 Ultrasound treatment

Extraction and formulation of peach‐based beverage are done before sonication treatments, following the method of Aadil, Zeng, Han, and Sun (2013). The sonication was performed at 20 kHZ frequency, and power was set at 130W (VC130, Sonics & Materials, Inc.). The ultrasound probe was held in a depth of 25 mm in the beverage to continuously sonicate it at different time durations (30, 60, and 90 min) by keeping temperature constant at 30 ± 5°C. The treated juice was stored in air tight and sterilized media bottles at −18°C for further analysis.

### 2.4 Determination of physicochemical attributes

#### 2.4.1 pH determination

The pH of peach beverage was measured according to the method illustrated by Lee, Durst, and Wrolstad (2005) using digital pH meter (HI2211‐Hanna Instruments Bedfordshire).

#### 2.4.2 Total soluble solid (Brix°) determination

Total soluble solid (°Brix) was measured by the method described by Kelebek, Selli, Canbas, and Cabaroglu (2009) using refractometer (Handheld Refractometer by VWR (0%–10% Brix)).

#### 2.4.3 Determination of cloud value

Cloud value of fruit beverage was determined by following the method outlined by Tiwari, Muthukumarappan, O'Donnell, and Cullen (2008) with some modification. Treated juice samples (1 ml each) were centrifuged at 1,025 *g* at 20°C for 10 min. Cloud value was measured as the supernatant absorbance using a Tecan Spark plate reader (Spark 10M, Serial # 1507006140) at 660 nm with distilled water serving as a blank.

### 2.5 Functional analysis

#### 2.5.1 Sample extraction for phenolic activity and antioxidant activity determination

The bioactive compounds and antioxidants were extracted from peach beverage following the methodology of Sun et al. (2013) with little modification. A 1 ml of peach‐mixed beverage was dissolved in 80% methanol to make a final volume of 10 ml in a falcon tube. The beverage mixture was then vortexed for 20 s and centrifuged at 3,500 *g* for 10 min, and resultant beverage mixture was then filtered using Whatman No.1 paper. Extracted sample was then kept at freezer at −18°C temperature until further analysis.

#### 2.5.2 Total phenolic content

The Folin–Ciocalteu procedure was used to determine the total phenol content (TPC) in peach‐mixed beverage as described by Canan et al. (2016) with some modification. The Folin reagent (150 µl), sample extract (30 µl), and 7.5% sodium carbonate solution (120 µl) were taken in a test tube and vortexed for 10 s. The mixture was then kept in incubator for 45 min at room temperature. The multimode plate reader (Tecan Spark 10M) was used to measure the absorbance of samples and blank (30 µl of 80% methanol + 150 µl of Folin reagent and 120 µl of sodium carbonate) at 765 nm, and the results were expressed as µg GAE/100 ml of juice.

#### 2.5.3 Total flavonoid content

To determine the flavonoid contents, the method of Liu (2008) was followed with some modification. Sample extract of 25 µl was added to each well containing 10 µl of 5% NaNO_3_ and 100 µl distilled water. The mixture was then left for 15 min at room temperature, and afterward, the 15 µl of 10% AlCl_3_ was added into the mixture. The final volume of mixture was attained after the addition of 1 M NaOH and 50 µl of distilled water into it. The resultant mixture was then kept in incubator for 1 hr, and multimode plate reader (Tecan Spark 10M) was used to measure the absorbance at 510 nm. The results are expressed as µg CE/100 ml of juice.

#### 2.5.4 Antioxidant activity

The antioxidant activity of mixed peach beverage was determined by three different methodologies: DPPH radical scavenging activity, ferric reducing antioxidant power (FRAP), and 2,2'‐azino‐bis(3‐ethylbenzothiazoline‐6‐sulfonic acid) (ABTS) radical cation method. The DPPH activity of peach beverage was examined according to the method illustrated by Tembo, Holmes, and Marshall (2017a), Tembo, Holmes, and Marshall (2017b) with slight modification. A 0.1 mM of 1,000 µl DPPH solution was poured in 25 µl of peach juice sample in methanol. After getting vortexed for 20 s, the mixture was then incubated for 15 min. Multimode plate reader (Tecan Spark 10M) was used to observe the absorbance of the sample and control (80% (v/v) methanol) at 517 nm. Results were expressed as % DPPH calculated as:

$$\%\text{DPPH}\;=\;(A_{C}-A_{S}/A_{C})\;\times \;100$$

where *A*_c_ is absorbance of control and *A*_s_ is of sample.

The (FRAP) assay was conducted following the methodology of Stratil, Klejdus, and Kubáň (2006) with little changes. A 187 µl FRAP reagent and 6 µl of peach‐mixed juice sample were added into falcon tubes and vortexed for 10 s. The resultant mixture was then incubated for 6 min. The beverage sample and blank (80% (v/v) methanol) were then placed in multimode plate reader (Tecan Spark 10M) to examine their absorbance at 593 nm. The values are expressed as µmol Trolox/L of juice.

The (ABTS) radical activity of peach beverage was carried out according to the method of Wojdyło, Figiel, and Oszmiański (2009) with slight changes. The ABTS^+^ aqueous solution (300 µl) was mixed with juice sample (10 µl), and for attaining the absorbance of 0.7 ± 0.02 at 734 nm, the mixed solution was diluted with deionized water. The samples were then incubated for 6 min, and after incubation, samples were placed in multimode plate reader (Tecan Spark 10M) to measure the absorbance of juice sample and blank (80% (v/v) methanol) at 734 nm. The values are expressed as µmol Trolox/L of juice.

### 2.6 Organic acid analysis by HPLC

Organic acids were extracted according to the methodology illustrated by Chebrolu, Jayaprakasha, Yoo, Jifon, and Patil (2012) with slight modification. A total volume of 100 ml of solution was developed by diluting peach juice sample with 3 g of metaphosphoric acid. The solution was then vortexed (20 s) and centrifuged (3,500 *g*, 10 min). The resultant mixture was then filtered using Whatman No.1 filter paper. The final filtration was done through PTFE filters (0.45 µm) before samples were poured into HPLC vials. The quantification of organic acids was done by UFLCXR HPLC equipment following the methodology illustrated by Pimpão et al. (2013). The HPLC vials were filled with 20 µl of juice sample. Potassium dihydrogen phosphate (10 mM) (pH 2.6) was used as a mobile phase, and Gemini C18 column (250 × 4.6 mm, 5 μm; Phenomenex) was used for the separations at 25°C. Isocratic conditions were applied for carrying out separation (0.5 ml/min; 15 min). The chromatograms of organic acids (malic, citric, oxalic, and shikimic) were recorded at 210 nm, and quantities are expressed as µg/100 ml of juice.

### 2.7 Statistical analysis

Results are expressed as means of at least three determinations of independent samples ± standard deviation (*SD*). ANOVA using least significant difference (LSD) (*p* ≤ .05) was performed to determine the significance of differences between treatments using IBM SPSS statistical software version 22. For multivariate data analysis, principal component analysis was applied according to physicochemical properties of peach juice.

## 3 RESULTS AND DISCUSSION

The modification in physicochemical and functional attributes of functional peach beverage was observed as follows after being exposed to conventional pasteurization and different ultrasound treatments.

### 3.1 pH

pH is one of the important factors as it is closely related to the stability of the bioactive compounds in fruits and fruits products (Sánchez‐Moreno, Plaza, de Ancos, & Cano, 2006). The pH value of functional beverage showed no significant differences (*p* > .05) under different pasteurization and ultrasound processing conditions (Table 1). The similar findings of sonication treatment having no effect on the pH value were observed in guava juice (Cheng, Soh, Liew, & Teh, 2007) and in blueberry juice (Zou & Hou, 2017). Low pH value of fruit juices also helps in preventing the growth of microbes in them. US processing held for 90 min gives more stability to pH (3.84 ± 0.015) in functional peach beverage than conventional pasteurization and other US treatment conditions.

**Table 1:** Effect of ultrasound processing on physicochemical parameters of functional peach beverage

Treatments	pH	TSS (°Brix)	CV
C	3.83 ± 0.005^abc^	13.8 ± 0.05^abc^	0.065 ± 0.0005^abcd^
CP	3.82 ± 0.005^abcd^	14.2 ± 0.15^abcd^	0.066 ± 0.0004^abc^
US_30_	3.83 ± 0.01^abcd^	13.83 ± 0.05^ab^	0.066 ± 0.0003^abcd^
US_60_	3.83 ± 0.005^abcd^	13.7 ± 0.05^abc^	0.0678 ± 0.0009^abcd^
US_90_	3.84 ± 0.015^abcd^	13.58 ± 0.05^ab^	0.070 ± 0.0007^abcd^

Note

Results are means of triplicates (±*SD*). Values with different letters in columns are significantly different (*p* ≤ .05).

Abbreviations: C, control; CP, conventional pasteurization; CV, cloud value; TSS, total soluble solid; US_30_, US_60_, and US_90_, ultrasound processed samples for 30, 60, and 90 min.

### 3.2 **Total soluble solids (Brix**°**)**

°Brix is one of the important factors for determining the quality of juices as it indicates the amount to soluble solids present in the juice. It is also an important component which stabilizes the bioactive compounds in juices (Zafra‐Rojas et al., 2013). The TSS (°Brix) value of formulated beverage showed the significant increment (*p* ≤ .05) in conventional pasteurization, whereas no significant change (*p* > .05) was observed in ultrasound processed juice samples (Table 1). Kumar, Khadka, Mishra, Kohli, and Upadhaya (2017) also observed that conventional pasteurization enhances the °Brix amount in pomelo juice. While according to the findings, the ultrasound processing did not affect the °Brix concentration of grapefruit juice (Aadil et al., 2013) and mango juice (Santhirasegaram, Razali, & Somasundram, 2013). The controlled °Brix amount is favorable in formulation of fruit juices as it highly affects the sensory attributes of product. Therefore, in our study, we conclude that ultrasound treatment is best option to maintain the total soluble contents of peach‐based beverage.

### 3.3 Cloud value

Cloud is composed of lipids, cellulose, protein, pectin, hemicellulose, and other minor components, and it is an important quality parameter that improves the flavor and color of fruit juices (Rajauria & Tiwari, 2017). The significant rise (*p* ≤ .05) in cloud value of functional peach beverage was observed in thermal pasteurization and ultrasound processing (Table 1). Similar rise in cloud index was seen by Aghajanzadeh and Ziaiifar (2018) with the increase in temperature up to 90°C and time up to 2.68 min in sour orange juice. The ultrasound processing increased the cloud value in grapefruit juice at different sonication timing due to the cavitation process which produces high pressure gradient and ultimately homogenize the juice (Aadil et al., 2015). The increment of cloud particles in juice by cavitation is also attributed to the breakdown of larger molecules that develops the suspended particles, thus improves the clouds in the juice (Iftikhar, Wagner, & Rizvi, 2014).

### 3.4 Total phenolic and total flavonoid contents

Phenolic compounds play a vital role in preventing and minimizing the risk of many degenerative and physiological diseases in human body, and therefore, their consumption is very essential and beneficial to human health (Apak, Özyürek, Güçlü, & Çapanoğlu, 2016). The total phenolic and total flavonoid contents of functional peach beverage showed the significant decrement (*p* ≤ .05) in thermal pasteurization treatment and significant increment (*p* ≤ .05) in all sonicated treatments (Table 2). The similar effect of pasteurization decreasing the total phenolic and total flavonoid contents was observed in baobab (Tembo et al., 2017a, 2017b) and citrus (Brasili et al., 2017) juices. On the other hand, the increasing trend of TPC and TFC contents with ultrasound processing was observed in different studies on blueberry juice (Zou & Hou, 2017), mango juice (Santhirasegaram et al., 2013), watermelon and carambola juices (Saikia, Mahnot, & Mahanta, 2016), and grapefruit juice (Aadil et al., 2013).

**Table 2:** Effect of ultrasound processing on phenolic profile and antioxidant activity of functional peach beverage

Treatments	TPC (µg GAE/100 ml)	TFC (µg CE/100 ml)	DPPH (%)	FRAP (µmol Trolox/L)	ABTS (µmol Trolox/L)
C	432.09 ± 0.0004^abcd^	169.5 ± 0.0003^ab^	42.43 ± 0.06^abcd^	398.13 ± 0.007^abcd^	1,403.25 ± 0.007^abcd^
CP	418.19 ± 0.0005^abcd^	167.5 ± 0.0002^abc^	40.12 ± 0.01^ns^	363.63 ± 0.009^abcd^	1,257.87 ± 0.008^abcde^
US_30_	482.716 ± 0.0002^abcd^	172.5 ± 0.0003^a^	48.11 ± 0.03^ns^	458.63 ± 0.004^abcd^	1,455.875 ± 0.005^abcd^
US_60_	531.48 ± 0.0002^abcd^	173 ± 0.0001^ab^	50.36 ± 0.02^a^	472.53 ± 0.004^abcd^	1,471.25 ± 0.007^abcd^
US_90_	600.61 ± 0.0002^abcd^	177 ± 0.0004^abcd^	51.87 ± 0.01^a^	506.13 ± 0.0009^abcd^	1507.375 ± 0.001^abcd^

Note

Results are means of triplicates (±*SD*). Values with different letters in columns are significantly different (*p* ≤ .05).

Abbreviations: C, control; CP, conventional pasteurization; US_30_, US_60_, and US_90_, ultrasound processed samples for 30, 60, and 90 min.

The increase in phenolic contents by ultrasound treatment might be because the exertion of cavitation pressure leads the breakdown of cell wall releasing the phenolic compounds present there in bounded form (Chen et al., 2013). Phenolics are among the major compounds that maintain the quality of fruit juices, and therefore, it is highly recommended to enhance or preserve them while going through different processing techniques. In our study, the best processing condition that enhances the TPC (600.61 ± 0.0002 µg GAE/100 ml) and TFC (177 ± 0.0004 µg CE/100 ml) contents of formulated peach‐based beverage is ultrasound held for 90 min as compared to control and other treatments.

### 3.5 Antioxidant activity

The impact of conventional thermal pasteurization and ultrasound processing techniques was studied on antioxidants of functional beverage by different analysis (DPPH, FRAP, ABTS). The antioxidant activity analyzed by different methods showed significant differences (*p* ≤ .05) under thermal and nonthermal processing conditions (Table 2). The conventional pasteurization showed the decreasing trend in all three antioxidant tests (DPPH, FRAP, and ABTS). On the other hand, the antioxidants keep on increasing with the increase in sonication treatment time. The similar effect of ultrasound processing was determined on the antioxidant activity of purple cactus pear juice with DPPH, ABTS, and FRAP methodology at different amplitude and time durations (Zafra‐Rojas et al., 2013).

According to the findings, the high level of antioxidants was observed in samples when treated with maximum amplitude and longest time duration. The increase in antioxidant activity in mango juice with time was also described by Santhirasegaram et al. (2013). Phenolic compounds are one of the main contributors in antioxidant activity in fruit juices. Previous study shows that there is a positive relation between total phenolic contents and antioxidant activity in many plant species (Duh, Yen, Yen, Wang, & Chang, 2004; Jayaprakasha, Girennavar, & Patil, 2008). Therefore, increase or decrease in antioxidant activity is directly proportional to loss or gain of phenolic compounds in the juices.

### 3.6 Organic acids

**Figure 1:**
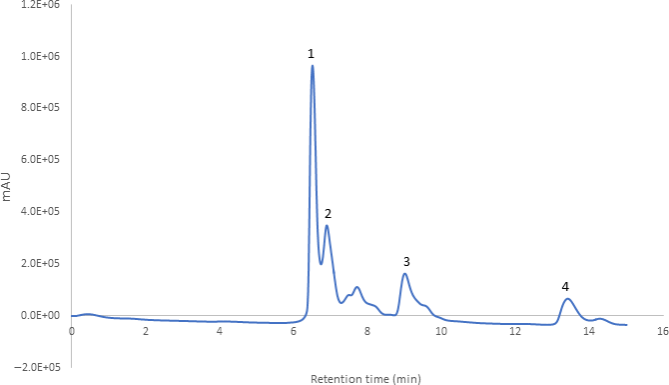
HPLC chromatogram of organic acids at 210 nm (1. malic acid, 2. citric acid, 3. oxalic acid, and 4. shikimic acid)

The effects of conventional thermal pasteurization and ultrasound processing were examined on different organic acids present in functional peach beverage and were screened using HPLC for the presence of organic acids (Figure 1). Organic acids present in formulated beverage (malic, citric, oxalic, and shikimic acids) showed significant changes (*p* ≤ .05) under conventional thermal and ultrasound processing conditions (Table 3).

**Table 3:** Effect of ultrasound processing on organic acids of functional peach beverage

Treatments	MA (µg/100 ml)	CA (µg/100 ml)	OX(µg/100 ml)	SH (µg/100 ml)
C	905 ± 20.1^abcd^	81 ± 0.7^abcd^	112 ± 1.2^abcd^	42 ± 0.8^abcd^
CP	876 ± 15.8^abcd^	59 ± 0.6^abcd^	88 ± 1.4^abcd^	28 ± 0.6^abc^
US_30_	949 ± 14.6^abc^	102 ± 0.6^abcd^	131 ± 1.3^abcd^	48 ± 0.4^ab^
US_60_	987 ± 13.8^abcd^	115 ± 0.4^abcd^	139 ± 1.2^abcd^	60 ± 0.3^ab^
US_90_	998 ± 15.3^abcd^	128 ± 0.8^abcd^	145 ± 1.5^abcd^	63 ± 0.5^abcd^

Note

Results are means of triplicates (±*SD*). Values with different letters in columns are significantly different (*p* ≤ .05).

Abbreviations: C, control; CA, citric acid; CP, conventional pasteurization; MA, malic acid; OX, oxalic acid; SH, shikimic acid; US_60_, US_30_, and US_90_, ultrasound processed samples for 30, 60, and 90 min.

The conventional pasteurization processing showed the significant fall in the concentration of organic acids in functional beverage, whereas the ultrasound processing technique has the positive impact in increasing the organic acid concentration in beverage. This beneficiary effect of ultrasound in increasing the amount of organic acids was also reported by Aadil et al. (2013) in grapefruit juice. The increase in organic acid concentration after ultrasound processing might be due to removal of entrapped oxygen due to cavitation pressure (Cheng et al., 2007). The ultrasound processing for 90 min is referred as a best treatment that improves the amount of organic acids (MA = 998 ± 15.3; CA = 128 ± 0.8, OX = 145 ± 1.5; SH = 63 ± 0.5 µg/100 ml) in functional peach‐based beverage as compared to control.

### 3.7 Principal component analysis

**Figure 2:**
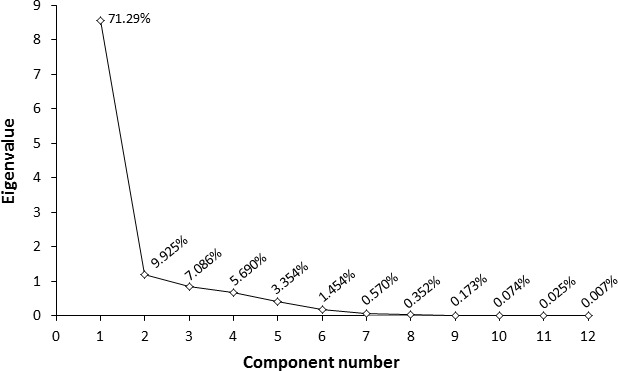
Eigenvalues of each principal component for ultrasound processed peach juice

Multivariate data analysis was done by principal component analysis (PCA) because it can explain the original data which help to characterize the quality of juices (Jolliffe & Cadima, 2016). PCA is a method to identify patterns in data by comparing data sets which is further expressed based on their similarities and discrimination, respectively (Ahmad, Nache, Hinrichs, & Hitzmann, 2016). According to Figure 2 which explains total variance in PCA of sonicated juice, it could be seen that largest eigenvalue is of principal component 1 (PC1: 8.555) with the variance of 71.29% and principal component 2 (PC2: 1.191) with the variance of 9.925%, respectively, whereas PC3 to PC12 yielded smaller amount of eigenvalues which is <1, and therefore according to Kaiser's rule, these components cannot be considered for further studies. Thus, the original 12 set data were compressed to 2 set data that explained 81.285% of the variances of all measured physicochemical properties of peach juice.

**Figure 3:**
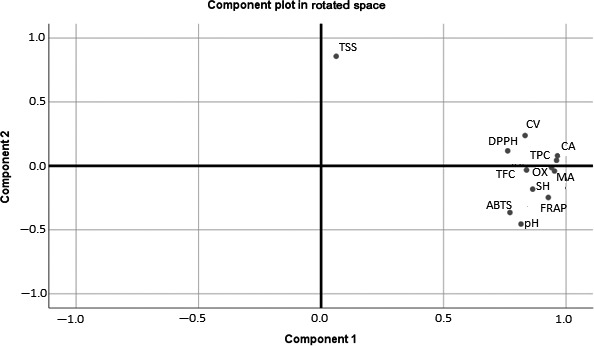
Principal component plot (PC1 × PC2) in rotated space for ultrasound processed peach juice

Figure 3 represents the loading variables of PCA in first two PCs which represents the correlation between the juice components and tested physicochemical properties. It is deduced that PC1 was negatively correlated with total soluble solid (TSS) and positively correlated with all other properties, whereas PC2 is negatively correlated with pH, FRAP, ABTS, and Shikimic acid and positively correlated with TSS, TPC, TFC, DPPH, Cloud value, and other organic acids. Samples which lie close to each other in score plot exhibits high similarity in juice quality. The observation from PCA figures is according to the data summarized in original data of mean value (Tables 1, 2, 3).

## 4 CONCLUSION

The effect of conventional pasteurization and ultrasound processing was examined on the physicochemical parameters, total phenolic and total flavonoid contents, antioxidant activity, and organic acids of functional peach‐based beverage under different processing conditions. Conventional pasteurization causes the decline in quality parameters of functional juice, whereas the significant increment in bioactive compounds, antioxidant activity, organic acid concentration, and clarity was observed in juice samples after being exposed to sonication treatment for 90‐min duration. Thus, ultrasound treatment is good alternative to thermal treatment in beverage industry that could maintain the quality far superior than conventional pasteurization. Further research work is needed to develop models to optimize the processing variables during sonication treatments in in‐line system as well to make this technique compatible with existing setup of industrial pasteurization.

## CONFLICT OF INTEREST

The authors declare no conflict of interest.

## ETHICAL APPROVAL

This study does not involve any human or animal testing.
